# Effect of Pretreatment on Biomass Residue Structure and the Application of Pyrolysed and Composted Biomass Residues in Soilless Culture

**DOI:** 10.1371/journal.pone.0064550

**Published:** 2013-05-21

**Authors:** Linna Suo, Xiangyang Sun, Weijie Jiang

**Affiliations:** 1 College of Soil and Water Conservation, Beijing Forestry University, Beijing, P.R. China; 2 The Institute of Vegetables and Flowers, Chinese Academy of Agricultural Sciences, Beijing, P.R. China; Roehampton University, United Kingdom

## Abstract

The changes in the structural characteristics of biomass residues during pyrolysis and composting were investigated. The biomass residues particles were prepared by pyrolysing at temperatures ranging from 350 to 400. For soilless production of the ornamental plant Anthurium andraeanum, pure sphagnum peat moss (P) has traditionally been used as the growing medium. This use of P must be reduced, however, because P is an expensive and nonrenewable resource. The current study investigated the use of biomass residues as substitutes for P in A. andraeanum production. Plants were grown for 15 months in 10 soilless media that contained different proportions of pyrolysed corn cobs (PC), composted corn cobs (C), pyrolysed garden wastes (PG), and P. Although the media altered the plant nutrient content, A. andraeanum growth, development, and yield were similar with media consisting of 50% P+50% PC, 50% P+35% PC+15% PG, and 100% P. This finding indicates that, when pyrolysed, organic wastes, which are otherwise an environmental problem, can be used to reduce the requirement for peat in the soilless culture of A. andraeanum.

## Introduction

Anthurium (*Anthurium andraeanum*) is one of the most important of the tropical cut flowers [1]. Although anthurium was introduced to the Chinese market by flower exporters, particularly of Dutch origin [2], Chinese horticulture is developing rapidly, and the flower is now produced in greenhouses in Beijing, Taiwan, and Yunnan province in China [2]–[7]. With the increase in the transfer of knowledge about cultivation of anthurium, the quality of locally cultivated products is improving rapidly [2].

Anthurium and other soilless cultivated flowers are commonly grown in a peat medium, and China imports large quantities of peat for horticultural production. The continued use of peat is problematic, however, because it is an expensive and non-renewable resource [8], i.e., supplies of peat are limited, and the collection of peat raises environmental concerns [5]–[7]. If properly composted, a number of organic residues can be used as growth media in place of peat; these include urban solid wastes, sewage sludge, pruning waste, spent mushroom, and even green wastes [9]–[12].

To grow well, anthurium requires a medium that has ample porosity (to provide sufficient space for root growth and good gas exchange), a low salt content, and a slightly acidic pH [2]. Because the addition of municipal solid waste (MSW) compost has an alkalinizing effect on soils [13], MSW is probably not useful for anthurium production. Porous carbon materials, in contrast, have attracted much attention as growth media because they have a large volume of very small pores and a high surface area [14]–[16]. One method for obtaining porous carbon materials is by pyrolysis of biomass wastes generated by agricultural activities.

Numerous studies have evaluated the use of compost made from waste material as a soilless medium, especially as an alternative to peat, for ornamental potted plants [17]–[19]. In contrast, only a few studies have focused on the use of pyrolysed biomass residues for the production of ornamental potted plants [20], [21], and no study has evaluated the use of pyrolysed biomass residues for the production of anthurium (*Anthurium andraeanum*) transplants.

The present study evaluated the suitability of pyrolysed and composted biomass residues mixed with conventional peat as growth media for the greenhouse production of anthurium. The effects of pretreatment on biomass residues characters and the media on growth and nutrient content of anthurium were evaluated.

## Materials and Methods

### Growing media and components

The materials used in this study included garden waste (branch and leaf litter) obtained from The Garden Waste Consumption Centre of Chaoyang District (Beijing, P.R. China), corn cobs obtained from a farm on the outskirts of Shijiazhuang (HB, P.R. China), and sphagnum peat moss (a long-fibred brown peat moss) that is commercially available as Pindstrup™ Substrate (Pindstrup Mosebrug A/S, Ryomgaard, Denmark). The first two materials were used to create the following three components: composted corn cobs (C), pyrolysed corn cobs (PC), and pyrolysed garden waste (PG). As a fourth component, sphagnum peat moss (P) was used without treatment.

For production of C, corn cobs were composted by the Daxing District Nursery (Beijing, P.R. China) using a 1∶100 (w/w) ratio of Wanguo™ fungi (Wanguo Biochemical Co. Ltd., Baoding, HB, P.R. China) and corn cobs. The corn cobs were screened to a maximum particle size of 20 mm. After the corn cobs and fungi had been incubated for 10 weeks in an agitated bed system, with interior temperatures reaching 55–77°C, the resulting corn cob compost was rescreened to 20 mm.

Pyrolysis of garden waste and corn cobs (not composted) was performed by the Tongzhou Shenlong Torch Company (Beijing, China) using an organic wastes pyrolysis reactor. Before pyrolysis, materials were cut into 10- to 20-mm particles and air-dried. The garden waste and corn cobs were pyrolysed at 350–400°C for 30 min [22]. After pyrolysis, all materials were placed in water at room temperature for 2 h at a material-to-water ratio of 1∶2 (v/v). The pyrolysed materials were then air-dried and passed through a 20-mm screen.

The four components were combined in different ratios (v/v) to produce the following 10 media: 100 C (meaning 100% C); 100PC; 100PG; 70∶30 C∶PG; 70∶30 PC∶PG; 50∶35∶15 P∶C∶PG; 50∶50 P∶PC; 50∶35∶15 P∶PC∶PG; 50∶50 P∶C; and a standard or control, which was 100P.

The physical and chemical characteristics of the 10 media were determined before and after the experiment described in the next section.

Electrical conductivity (EC) and pH were determined for a suspension containing a medium:water ratio of 1∶5 after the suspension had been shaken at 2.5 Hz in an end-over-end shaker for 2 h [23], [24]. The EC value was measured with a conductance meter (Model DDS-304; Shanghai Precision Instruments Co., Ltd., Shanghai, P.R. China). The pH was measured with a pH meter (Model 868; Thermo Fisher Scientific Inc., Waltham, MA, USA).

Total N contents were determined using a Kjeldahl total nitrogen apparatus (Model KDY-9830, Huawei Industrial Technology Co., Ltd., Beijing, P.R. China), and the concentrations of phosphorus, K, Ca, Mg, Na, Fe, Mn, Cu, and Zn were determined by inductively coupled plasma-atomic emission spectroscopy (Model AA-7000, Shanghai Precision Instruments Co., Ltd., Shanghai, P.R. China) [23].

Air content at water holding capacity and the water holding capacity of the 10 media were determined according to the method proposed by Guo (2003), and the bulk density, particle density, and total porosity were determined according to Bao (2000) [23], [24]. The air content at water holding capacity refers to the air-filled porosity at maximum water holding capacity of the media [24].

All physical and chemical properties were measured on three replicates of each medium, and the concentrations of each nutrient were expressed on a per weight basis (i.e., g kg^−1^ or mg kg^−1^).

Pore size distribution is one of the most important characteristics of porous materials with respect to the adsorption of organic compounds. The accessibility of the organic molecules to the microporosity depends on the pore size [25]. Pores are classified a micropores (<2 nm), mesopores (2–50 nm) and macropores (>50 nm). For scanning electron microscopy, the material was dehydrated in a series of ethanol and acetone solutions, critical point dried (EMS critical point drier), mounted on stubs, and coated with gold in a sputter coater. Specimens were examined in a scanning electron microscope (Hitachi S-3400 N).

### Plant material and growing conditions

An experiment was conducted in an 800-m^2^ plastic greenhouse at the Daxing District Nursery in Beijing, P.R. China, from November 2008 to April 2010. The greenhouse was maintained at 20–24°C.

Tissue-cultured plantlets of *A. andraeanum* ‘Pink Lady’ were used and were 16–18 cm tall with an average of 5.2 leaves at the start of the study. On 26 November 2008 (month 0), 300 plants were randomly selected and transplanted (bare root) into 14-cm-diameter plastic pots (one plantlet per pot) containing 1.0 L of one of the 10 media. The 30 pots for each medium were arranged in a randomized complete block design with 10 pots per treatment per block. The plants were immediately irrigated to saturation.

The plants were irrigated from below the leaf crown with nutrient solution two times per week using a hose and a mist nozzle connected to a water control system. Each plant received approximately 400 ml of nutrient solution (pH 5.7, EC 1.2 mS cm^−1^) each time. The content of nutrients (mg/l) in the solution was: N-NH_4_^+^ (7), N-NO_3_^−^ (112), phosphorus (47), K (196), Ca (112), Mg (19), Fe (0.84), Mn (0.17), B (0.11), Zn (0.2), Cu (0.05), and Mo (0.08).

### Measurement of plant growth, development, and yield

Shoot height (SH), leaf number (NL), and crown width (CW) were recorded at the beginning of the experiment (month 0). At the end of the experiment (month 15), NL, SH, CW, leaf area, flower number (NF), peduncle length (PL), spathe length, and spathe width per plant were determined for all plants [26].

Shoot heights (SH) of the plants were measured from the stem base to the tip of the largest leaf [27].

Flower size (FS)  =  (spathe length + width)/2 [27].

Leaf area was measured with a Licor LI-3000 portable leaf area meter. Chlorophyll was extracted from the leaf tissue by immersion in dimethylformamide (50 ml/g FW) for 48 h at 4°C. Chlorophyll a and b contents were determined as described by Arnon (1949).

At the end of the experiment (month 15), all plants of each treatment were separated into shoot (S), root (R), and recently matured flowers (F) and destructively sampled. F included the spathe, spadix, and peduncle, and recently matured flowers were those with color change for 75% of flower length [3]. The dry weight and nutrient content (N, phosphorus, K, Ca, Mg, Na, Fe, Mn, Cu, and Zn) were measured [23], [27]. The nutrient content of each plant tissue sample was measured for three replicates.

The nutrient concentration in the whole plant was calculated as follows: Concentration  =  [(shoot dry weight × shoot nutrient concentration) + (root dry weight × root nutrient concentration)] ÷ (shoot dry weight + root dry weight) [3].

### Data analysis

For each parameter, the data from each of the 10 plants per replicate were averaged to obtain one value per replicate. The replicate means were then subjected to ANOVAs (n = 3). When an ANOVA was significant, treatment means were compared using the Duncan's multiple range test (*P*<0.05). All analyses were done using the SPSS statistical software package (SPSS 17.0, SPSS Inc., Chicago, Illinois, USA).

## Results

### Structural characterization of raw and treated materials

**Figure 1:**
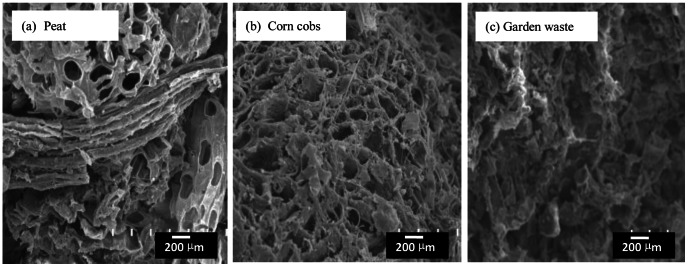
SEM micrographs (×200) of untreated (a) peat, (b) corn cobs, and (c) garden wastes.

**Figure 2:**
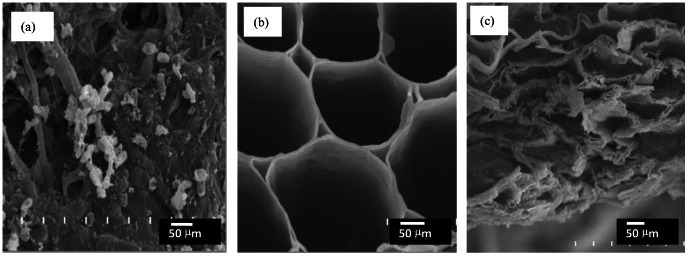
SEM micrographs (×1000) of the external surface of (a) composted corn cobs (×1000), (b) pyrolysed corn cobs (×1000), and (c) pyrolysed garden wastes.

Pore size distribution affects the kinetic properties of porous material and indicates the structural heterogeneity of porous materials SEM analysis shows the structure of the samples (Figure 1 and 2). Typical SEM photographs of peat and the raw waste materials (corn cobs and garden waste) are illustrated in Figure 1 (a)–(c). It was observed that peat gives both macropores like structure and fibrous like structure in nature with long ridges, resembling a series of parallel lines (Figure 1a). Otherwise, the raw corn cobs and garden waste give cross-interconnected pores spongy like (Figure 1b, c).

Different treatments caused noticeable changes in the structure of the waste materials. SEM micrographs of the external surfaces of the composted and pyrolysed samples are shown in Figure 2 (a)–(c). The composted corn cobs were covered by fungal hyphae (Figure 2a). In all pyrolysed samples, the external surfaces contained smooth, open pores of different sizes (Figure 2b, c). Furthermore, the pyrolysed corn cobs contained larger and more regular pores than the pyrolysed garden waste (Figure 2b, c). The external surface of pyrolysed corn cobs also had isodiametric, nearly circular cells with well-defined lumens arranged in a pattern that resembled a honeycomb (Figure 2b). The external surface of pyrolysed garden waste had an irregular, lamellar-like structure (Figure 2c).

### Growth media properties

At month 0, the physical properties of composted corn cobs (C), pyrolysed corn cobs (PC), and pyrolysed garden wastes (PG) were in almost all cases different from those of the control, which was Pindstrup sphagnum peat moss (P; Table 1). Water holding capacity was greater in P than in the other media, but the air content at water holding capacity was lower in P than in the other media (*P*<0.05; Table 1). Total porosity and bulk density in P+PC and P+PC+PG were not significantly different from total porosity and bulk density in P (Table 1).

**Table 1:** Physical and chemical properties of the 10 growing media at the beginning of the experiment.

Growing medium^a^	Air content at water holding capacity^b^ (% ,v/v)	Water holding capacity (% ,v/v)	Bulk density (g cm^−3^)	Particledensity (g cm^−3^)	Total porosity (%)	pH	EC (mS cm^−1^)
PC	30.5g^c^	62.1c	0.108ab	1.45a	92.6bc	6.70d	0.52c
C	28.3e	63.0d	0.172e	1.98i	91.3a	7.85h	1.02g
PG	32.7i	59.7a	0.133c	1.76f	92.4b	7.12f	0.69e
C+PG	29.7f	61.9bc	0.160d	1.91h	91.6a	7.55g	0.82f
PC+PG	31.4h	61.1b	0.115b	1.54c	92.5b	6.87e	0.57d
P+C+PG	24.3bc	68.1e	0.131c	1.73e	92.4b	6.60c	0.69e
P+PC	24.7cd	68.4e	0.104a	1.51b	93.1cd	6.49b	0.46b
P+PC+PG	25.2d	67.9e	0.107ab	1.55d	93.1cd	6.81e	0.48b
P+C	23.6b	68.8e	0.135c	1.77g	92.4b	6.80e	0.70e
P (control)	18.6a	75.0f	0.099a	1.55d	93.6d	5.65a	0.39a

a Composted corn cobs (C), pyrolysed corn cobs (PC), pyrolysed garden wastes (PG), and Pindstrup sphagnum peat moss (P); PC (100%), C (100%), PG (100%), C (70%) + PG (30%), PC (70%) + PG(30%), P (50%) + C (35%) + PG (15%), P (50%) + PC (50%), P (50%) + PC (35%) + PG (15%), and P (50%) + C (50%).

b The “air content at water holding capacity” indicates the air-filled porosity at maximum water holding capacity of the media [25].

c Within a column, means followed by different letters are significantly different according to Duncan's multiple range test at *P*<0.05 (n = 3).

At month 0, the pH was significantly lower in P than in all the other media (*P*<0.05; Table 1). The pH and EC were both significantly higher in the media containing C than in all other media (Month 0; *P*<0.05; Table 1).

The nutrient contents differed among the 10 media (Table 2).

**Table 2:** Nutrient concentrations of the 10 growing media at the beginning of the experiment.

Growing medium^a^	TKN^b^(g kg^−1^)	Phosphorus (g kg^−1^)	K (g kg^−1^)	Ca (g kg^−1^)	Mg (mg kg^−1^)	Zn (mg kg^−1^)	Fe (mg kg^−1^)	Mn (mg kg^−1^)
PC	0.120a^c^	0.042a	0.087i	0.041a	0.053b	169c	186b	101a
C	0.144bc	0.073f	0.044f	0.053b	0.048a	224h	179a	145h
PG	0.157cd	0.087h	0.024a	0.086i	0.069g	178e	220e	138f
C+PG	0.148bc	0.078g	0.039d	0.063e	0.054b	210g	191c	143g
PC+PG	0.132ab	0.058d	0.067h	0.055c	0.058c	171d	196d	112c
P+C+PG	0.162cd	0.065e	0.035c	0.070g	0.065e	179e	230h	121d
P+PC	0.148bc	0.052b	0.059g	0.061d	0.064e	158b	228g	101a
P+PC+PG	0.154cd	0.056c	0.049e	0.066f	0.067f	159b	233i	107b
P+C	0.156cd	0.064e	0.036c	0.065f	0.062d	185f	224f	124e
P (control)	0.172d	0.056c	0.027b	0.077h	0.075h	148a	270j	101a

a Composted corn cobs (C), pyrolysed corn cobs (PC), pyrolysed garden wastes (PG), and Pindstrup sphagnum peat moss (P); PC (100%), C (100%), PG (100%), C (70%) + PG (30%), PC (70%) + PG (30%), P (50%) + C (35%) + PG (15%), P (50%) + PC (50%), P (50%) + PC (35%) + PG (15%), and P (50%) + C (50%).

b TKN: Total Kjeldhal N.

c Within a column, means followed by different letters are significantly different according to Duncan's multiple range test at *P*<0.05 (n = 3).

Overall, the physical and chemical properties of the 10 media were within the recommended ranges for ornamental plant production in containers (Table 1, 2).

### Effects of media on nutrient uptake

#### N, phosphorus, and K

**Figure 3:**
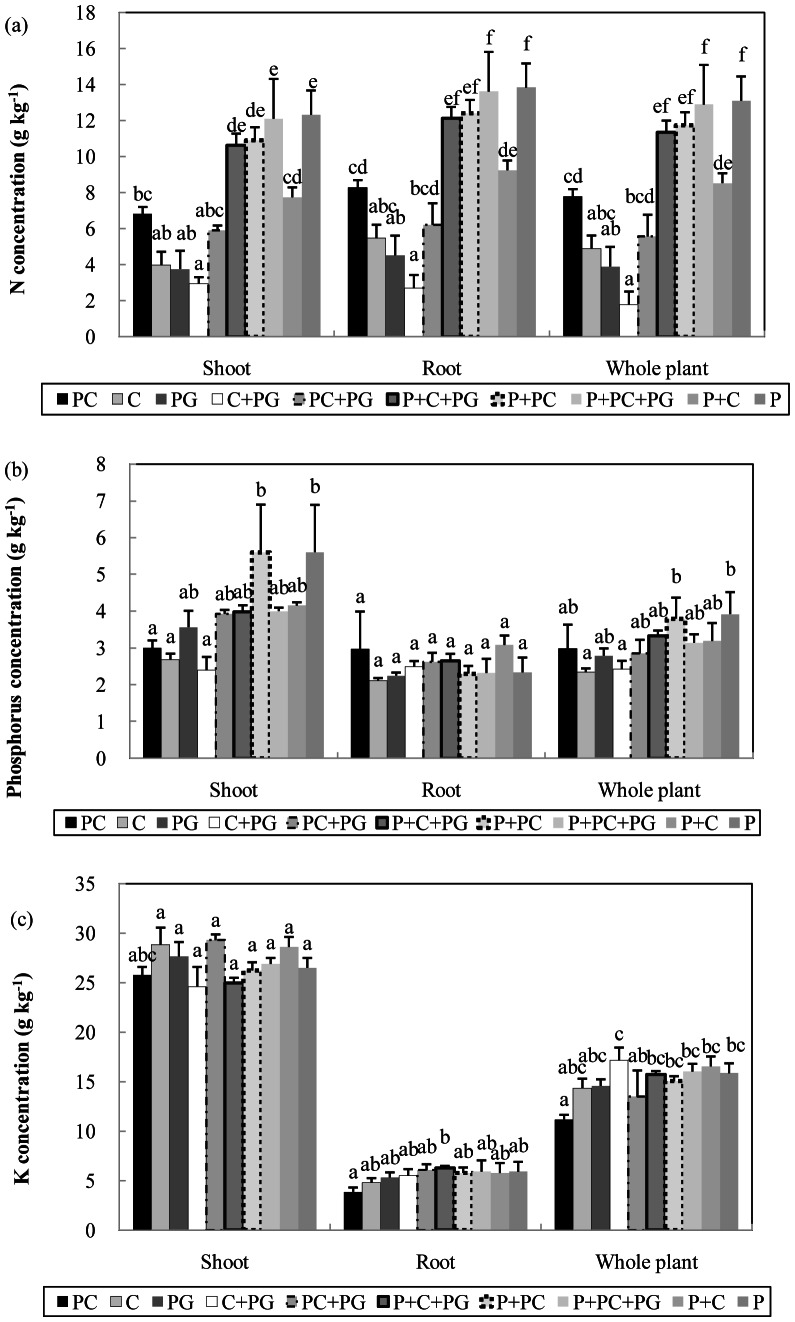
Effect of growing media on the concentration of (A) nitrogen, (B) phosphorus, and (C) potassium in *Anthurium andraeanum* cv. ‘Pink Lady’ at 15 months after planting. Composted corn cobs (C), pyrolysed corn cobs (PC), pyrolysed garden wastes (PG), and Pindstrup sphagnum peat moss (P); PC (100%), C (100%), PG (100%), C (70%) + PG (30%), PC (70%) + PG (30%), P (50%) + C (35%) + PG (15%), P (50%) + PC (50%), P (50%) + PC (35%) + PG (15%), and P (50%) + C (50%). Error bar denotes standard error of mean (n = 3). Means for the same plant part followed by the same letter are not significantly different according to Duncan's multiple range tests at *P*<0.05.

N concentrations in shoots, roots, and whole plants were significantly higher with media containing peat (except P+C) than with media lacking peat (PC, C, PG, C+PG, and PC+PG; *P*<0.01; Figure 3a).

The phosphorus concentrations in shoots were significantly lower with PC, C, and C+PG than with P+PC and P (*P*<0.01; Figure 3b). Phosphorus concentrations in roots did not significantly differ among the 10 media (Figure 3b). Phosphorus concentrations in whole plants were significantly higher with P+PC and P than with C and C+PG (*P*<0.05) but did not significantly differ among the other media (Figure 3b).

K concentrations in shoots were significantly higher with C and PC+PG than with C+PG and P+C+PG (*P*<0.05) but did not significantly differ among the other media (Figure 3c). K concentrations in roots were similar among most media but were significantly higher with P+C+PG than with PC (*P*<0.05; Figure 3c). K concentrations in whole plants were similar with most media but were significantly higher with C+PG than with PC (*P*<0.001; Figure 3c).

#### Ca, Mg, and Na

**Figure 4:**
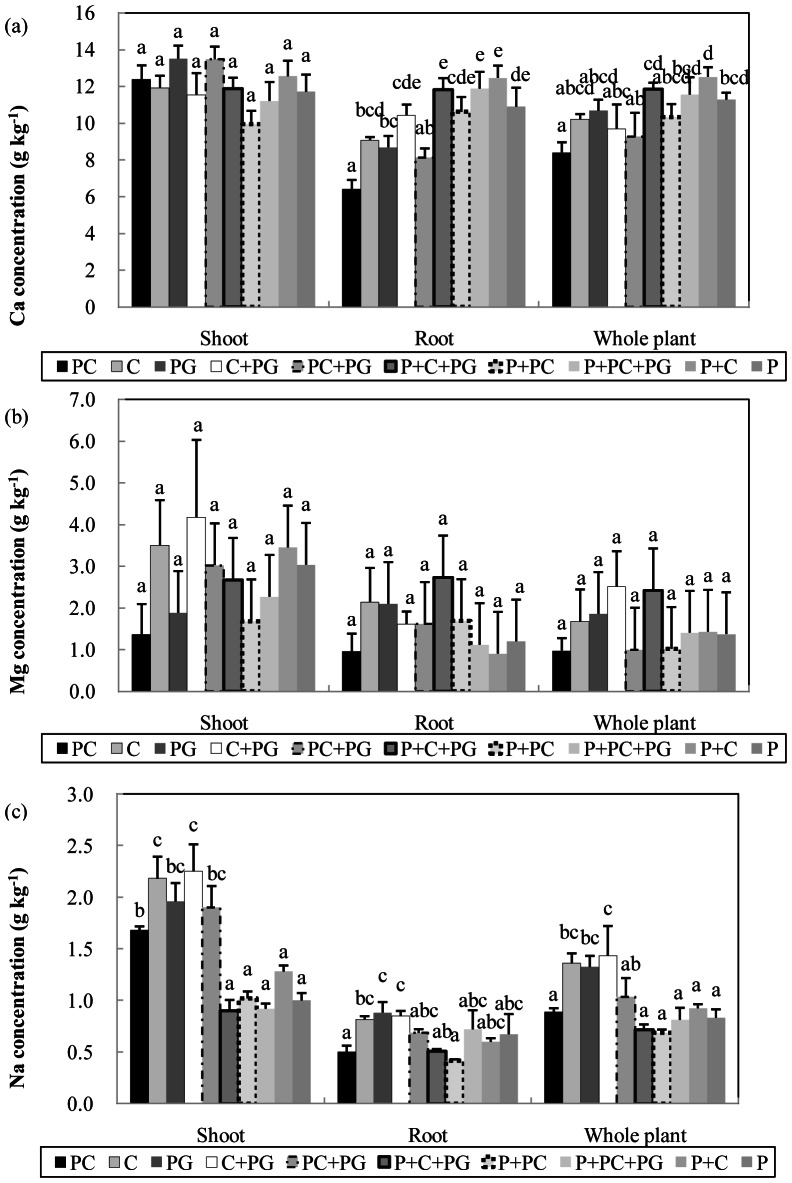
Effect of growing media on the concentration of (A) calcium, (B) magnesium, and (C) sodium in *Anthurium andraeanum* cv. ‘Pink Lady’ at 15 months after planting. Composted corn cobs (C), pyrolysed corn cobs (PC), pyrolysed garden wastes (PG), and Pindstrup sphagnum peat moss (P); PC (100%), C (100%), PG (100%), C (70%) + PG (30%), PC (70%) + PG (30%), P (50%) + C (35%) + PG (15%), P (50%) + PC (50%), P (50%) + PC (35%) + PG (15%), and P (50%) + C (50%). Error bar denotes standard error of mean (n = 3). Means for the same plant part followed by the same letter are not significantly different according to Duncan's multiple range tests at *P*<0.05.

The Ca concentrations in shoots were similar among most media but were significantly higher with PG and PC+PG than with P+PC (*P*<0.01; Figure 4a). Ca concentrations in roots were significantly lower with PC, PG, and PC+PG than with P (*P*<0.05) but did not differ between the other media and P (the control; Figure 4a). Ca concentrations in whole plants were significantly higher with P+C+PG and P+C than with PC and PC+PG (*P*<0.05) but did not differ among the other media (Figure 4a).

Mg concentrations in whole plants and in plant parts did not significantly differ among the 10 media (*P*>0.05,Figure 4b).

Na concentrations in shoot were significantly lower with media containing peat (P+C+PG, P+PC, P+PC+PG, P+C, and P) than with media lacking peat (*P*<0.05, Figure 4c). Na concentrations in roots were significantly higher with PG and C+PG than with PC and P+PC (*P*<0.05) but did not significantly differ among the other media (Figure 4c). Na concentrations in whole plants were significantly higher with C, PG, and C+PG than with the other media (*P*<0.01) except PC+PG (Figure 4c).

#### Micronutrients

Fe concentrations in leaves tended to be highest with PC and lowest with P+C+PG and P+PC+PG (Table 3). Mn concentrations in leaves tended to be highest with PC, PG, and PC+PG (Table 3). Cu concentrations in leaves tended to be lowest with C and C+PG (Table 3). Zn concentrations in leaves tended to be highest with PC+PG and lowest with P+PC (Table 3).

**Table 3:** Micronutrient content (mg kg^−1^) of leaves at the end of the experiment as affected by growing medium.

Growing medium^a^	Fe	Mn	Cu	Zn
PC	69.4c^b^	107bc	3.5ab	81de
C	58.0bc	72a	2.2a	42ab
PG	48.8abc	111c	5.4c	62bcd
C+PG	51.6abc	79a	3.2a	38ab
PC+PG	47.0abc	114c	5.2bc	95e
P+C+PG	33.6a	91ab	6.4c	44ab
P+PC	35.7ab	73a	6.5c	36a
P+PC+PG	29.9a	75a	6.6c	39ab
P+C	45.9abc	80a	6.8c	68cd
P(control)	40.4ab	73a	7.0c	50abc
*Sig.*	*	**	**	**

a Composted corn cobs (C), pyrolysed corn cobs (PC), pyrolysed garden wastes (PG), and Pindstrup sphagnum peat moss (P); PC (100%), C (100%), PG (100%), C (70%) + PG (30%), PC (70%) + PG (30%), P (50%) + C (35%) + PG (15%), P (50%) + PC (50%), P (50%) + PC (35%) + PG (15%), and P (50%) + C (50%).

b Within a column, means followed by different letters are significantly different according to Duncan's multiple range test at *P*<0.05. The last row indicates the significance of medium according to the ANOVA:

*P<0.05;

**P<0.01 (n = 3).

Fe, Mn, and Zn concentrations in leaves were all significantly higher with PC than with P (the control, Table 3). However, Cu concentration in leaves was significantly lower with PC than with P (Table 3).

### Effects of media on plant development and yield

Shoot dry weight were significantly lower with C, PG, and PC+PG than with P (*P*<0.05) but did not differ between the other media and P (the control; Table 4).

**Table 4:** Effects of growing medium on shoot dry weight (SM), shoot height (SH), crown width (CW), number of leaves (NL), root: shoot ratio (R∶S), peduncle length (PL), flower size (FS), and flower number per plant (NF) at the end of the experiment.

Growing medium^a^	SM (g/plant)	SH (cm)	CW (cm)	NL (number /plant)	R∶S	PL (cm)	FS (cm)	NF (number /plant)
PC	29.3abc^b^	28.3ab	32.0a	55.2ab	0.67c	18.2	5.1	4.2
C	24.1a	26.9ab	31.9a	59.0b	0.60bc	17.4	5.7	3.3
PG	26.4ab	26.7ab	31.4a	51.0ab	0.59bc	18.6	5.4	4.6
C+PG	42.3d	25.7a	31.8a	44.0a	0.40a	18.1	4.6	2.0
PC+PG	24.3a	29.4bc	31.7a	48.2ab	0.57bc	17.8	5.1	3.8
P+C+PG	39.5cd	37.5f	37.5b	54.4ab	0.50ab	18.4	5.4	3.2
P+PC	36.7bcd	36.1ef	37.4b	54.4ab	0.54b	20.5	5.8	5.2
P+PC+PG	39.9cd	32.0cd	37.3b	47.8a	0.52ab	19.4	5.8	4.7
P+C	29.9abc	33.6de	34.6ab	45.4a	0.52ab	15.4	5.9	4.0
P(control)	38.2cd	33.8de	37.3b	50.0ab	0.51ab	19.6	5.7	4.6
*Sig.*	**	**	**	*	**	n.s.	n.s.	n.s.

a Composted corn cobs (C), pyrolysed corn cobs (PC), pyrolysed garden wastes (PG), and Pindstrup sphagnum peat moss (P); PC (100%), C (100%), PG (100%), C (70%) + PG (30%), PC (70%) + PG(30%), P (50%) + C (35%) + PG (15%), P (50%) + PC (50%), P (50%) + PC (35%) + PG (15%), and P (50%) + C (50%).

b Within a column, means followed by different letters are significantly different according to Duncan's multiple range test at *P*<0.05. The last row indicates the significance of medium according to the ANOVA: n.s.  =  not significant;

*P<0.05;

**P<0.01 (n = 3).

Shoot height and crown width were lower in media lacking peat (PC, C, PG, C+PG, and PC+PG) than in media with peat (*P*<0.05, Table 4).

The number of leaves (NL) tended to be highest with C and lowest with C+PG, P+PC+PG, and P+C (*P*<0.05, Table 4). But there was no significantly difference between P (the control) and the other media (Table 4).

The root:shoot ratio (R∶S, based on dry weight) was significantly higher with PC than with P (*P*<0.05) but did not differ between the other media and P (the control; Table 4).

There were no significant differences in peduncle length (PL), flower size (FS), and flower number per plant (NF) among the 10 treatments at the end of the experiment (Table 4).

**Figure 5:**
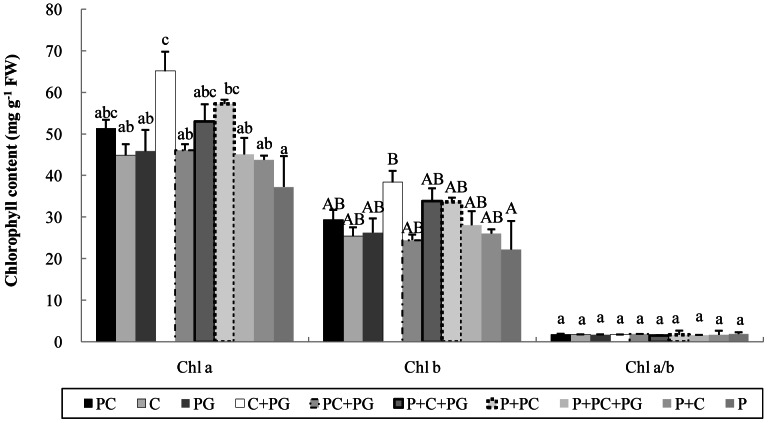
Chlorophyll a and chlorophyll b content (mg g^−1^ FW) and chlorophyll a/b ratio in *Anthurium andraeanum* cv. ‘Pink Lady’ after growing for 15 months in 10 media. Composted corn cobs (C), pyrolysed corn cobs (PC), pyrolysed garden wastes (PG), and Pindstrup sphagnum peat moss (P); PC (100%), C (100%), PG (100%), C (70%) + PG (30%), PC (70%) + PG (30%), P (50%) + C (35%) + PG (15%), P (50%) + PC (50%), P (50%) + PC (35%) + PG (15%), and P (50%) + C (50%). Error bar denotes standard error of mean (n = 3). Means for each kind of chlorophyll followed by the same letter are not significantly different according to Duncan's multiple range tests at *P*<0.05.

Chlorophyll content was significantly higher with C+PG than with P (*P*<0.05) but did not differ between the other media and P (the control; Figure 5).

## Discussion

This study investigated the effects of pyrolysed and composted biomass residues on the growth and flowering of anthurium (*A. andraeanum* ‘Pink Lady’). Although substantial information is available concerning the effects of composts on plant growth, little information is available concerning the effects of pyrolysed biomass residues on anthurium growth and flowering [4], [10], [17], [28].

Given the irrigation regime and the water holding capacity of the media (Table 1), water availability was unlikely to have limited growth of *A. andraeanum* in this experiment [26].

### Effects of different pretreatments

The physical and chemical characters of the studied biomass residues were more stable after pretreatment. In addition, air content at water holding capacity was higher for the pyrolysed materials than for raw materials and composted materials. However, the water holding capacity was higher for composted materials than for raw materials and pyrolysed materials.

### Effects of pH and electrical conductivity

*A. andraeanum* is salt-sensitive and prefers a slightly acidic pH [2]. The pH values of media containing P or PC were slightly acidic and were lower than in the media containing C or PG (Table 1). Micronutrients and metal cations are most soluble and available for plant uptake under acidic conditions [29].

Electrical conductivity (EC) is often used as a measure of the salt content of soil or growing media [29]. Plants are harmed by excess salts, and Na can damage media structure [13]. In the current study, the EC values were higher in media containing C than in media containing P or PC (Table 1). However, the EC values of the 10 media were within the recommended range for *A. andraeanum* growth and should not have limited effect on the plant (Table 1).

### The correlation between nutritional status and physiological response of A. *andraeanum*

*A. andraeanum* requires an adequate N and K supply to grow and to flower: as the supply of these nutrients decreases, time until flowering increases [30]. For corn and ryegrass, MSW compost is a poor source of N [31], [32]. The retarded growth of *A. andraeanum* in media lacking peat and in the P+C medium apparently resulted from an insufficient supply of N, as indicated by a reduced N concentration in the plant tissue (Figure 3a).

The ability of the media to supply phosphorus, K, Ca, and Mg was evidently not a limiting factor for plant growth in the current study.

The higher concentrations of Na in plants growing in media without peat than with peat may be related to the lower shoot height and smaller crown width of plants growing without peat. If Na uptake was not commensurately reduced, a reduced biomass would have resulted in an increased concentration of Na.

According to previous reports, adequate concentrations of Fe, Mn, Cu, and Zn in mature leaves *A. andraeanum* range from 25.5–70.5, 44.0–195.0, 4.0–14.0, and 17.0–87.4 mg kg^−1^, respectively [33]–[35]. In *A. andraeanum* in the current study, the concentrations of these four elements were all within these ranges.

Although the medium affected the concentration of nutrients in *A. andraeanum*, medium did not affect flower yield and size in the current study or in a previous study [3]. These findings suggest that the nutrients supplied by all the media in the present study were adequate for producing quality flowers.

The higher root:shoot ratio (R∶S) of plants in media containing PC may be attributed to the lower pH value and the superior aeration provided by these media.

## Conclusion

The growth, development, and yield of *A. andraeanum* were similar whether the plants were grown in P+PC medium, P+PC+PG medium, or in pure peat (P, the control).These findings, which indicates that P+PC and P+PC+PG can be used in place of peat for the production of cut flowers of *A. andraeanum* under soilless conditions, are significant because peat is non-renewable and expensive. The use of these replacement media, which contain a mixture of peat and pyrolysed biomass residues, would reduce (but not eliminate) the requirement for peat in soilless culture. In addition, the use of pyrolysed biomass in growing media provides another way to convert biomass residues into useful products.
